# *C9ORF72* and the FTD-ALS spectrum: A systematic review of neuroimaging studies

**DOI:** 10.1590/1980-57642015DN94000413

**Published:** 2015

**Authors:** Laura de Godoy Rousseff Prado, Isabella Carolina Santos Bicalho, Daiane Magalhães, Paulo Caramelli, Antônio Lúcio Teixeira12, Leonardo Cruz de Souza

**Affiliations:** 1 Postgraduate Program of Neuroscience, Universidade Federal de Minas Gerais (UFMG), Belo Horizonte, MG, Brazil.; 2 Neuromuscular Diseases Center, Department of Neurology, University Hospital, UFMG.; 3 Universidade José do Rosário Vellano – UNIFENAS, Belo Horizonte, MG, Brazil.; 4 Internal Medicine Department, Medical School, UFMG.; 5 Department of Neurology - University Hospital, UFMG.

**Keywords:** amyotrophic lateral sclerosis, frontotemporal dementia, *C9ORF72* repeat expansion, neuroimaging, esclerose lateral amiotrófica, demência frontotemporal, expansão *C9ORF72*, neuroimagem

## Abstract

**Objective:**

To perform a systematic review of the literature on the neuroimaging investigation of frontotemporal dementia (FTD) and amyotrophic lateral sclerosis (ALS) associated with *C9ORF72* mutation.

**Methods:**

The search was performed on PubMed and LILACS with the following terms: *C9ORF72*, MRI, SPECT, PET, ALS, FTD. No filters were added.

**Results:**

Twenty articles were selected. Most studies found consistent involvement of frontotemporal regions in *C9ORF72* carriers, including prefrontal cortex, and also cingulate, subcortical regions, especially the thalami, and posterior regions such as the parietal and occipital lobes. Functional connectivity was also explored and impaired sensorimotor connectivity in striatum and thalami was found in behavioral variant FTD *C9ORF72* carriers. Some papers have reported an absence of significant abnormalities on brain imaging.

**Conclusion:**

The inclusion of patients at different stages of the disease, differences in neuroimaging methods across studies, and distinct clinical phenotypes associated with *C9ORF72* may account for the heterogeneity of results.

## Abstract (pt)

**Objetivo:**

Realizar uma revisão sistemática da literatura sobre os estudos de neuroimagem da demência frontotemporal (DFT) e esclerose lateral amiotrófica (ELA), associadas à mutação *C9ORF72*.

**Métodos:**

A pesquisa foi realizada nas bases PubMed e LILACS com os seguintes termos: *C9ORF72*, MRI, SPECT, PET, ALS, FTD. Nenhum filtro foi utilizado.

**Resultados:**

Vinte artigos foram incluídos. A maioria dos estudos encontrou, nos portadores da expansão *C9ORF72*, envolvimento significativo das regiões frontotemporais, incluindo o córtex pré-frontal e também o cíngulo, regiões subcorticais (especialmente o tálamo) e regiões posteriores, como os lobos parietal e occipital. A conectividade funcional também foi investigada e disfunção sensório-motora foi demonstrada no estriado e no tálamo em pacientes com a variante comportamental da DFT associada à expansão *C9ORF72*. Alguns trabalhos não evidenciaram alterações significativas na neuroimagem.

**Conclusão:**

A inclusão de pacientes em diferentes estágios da doença, a variabilidade dos métodos de neuroimagem utilizados nos estudos e os distintos fenótipos de *C9ORF72* podem contribuir para a heterogeneidade dos resultados.

## INTRODUCTION

Frontotemporal dementia (FTD) and amyotrophic lateral sclerosis (ALS) share common clinical, pathological and genetic features. FTD encompasses a heterogeneous group of clinical presentations, with variable phenotypes including behavioral changes and deficits in language and other cognitive functions.^1,2^ On the other hand, besides motor symptoms, ALS is also characterized by cognitive impairment and behavioral disorders, overlapping with the cognitive profile of FTD.^3^ Indeed, the association between dementia and ALS has been recognized since the nineteenth century and almost 50% of ALS patients are believed to have cognitive impairment and up to 15% of these fulfill criteria for FTD.^3,4^ Conversely, motor neuron disease can appear during the course of FTD in up to 15% of patients.^5^ Therefore there is a clinical and pathophysiological continuum between FTD and ALS.

The recent discovery that an expanded hexanucleotide (GGGGCC) repeat insertion in a noncoding promoter region of open-reading frame 72 (*C9ORF72*) is a cause of familial FTD and ALS opened a promising window for the understanding of the FTD-ALS spectrum.^6,7^ The neurobiological functions of *C9ORF72* and the pathophysiological mechanisms by which it participates in neurodegenerative processes are unknown.^8^ The *C9ORF72* genotype may account for 10-50% of familial cases of behavioral variant FTD (bvFTD).^1,8^ Conversely, up to 41% of familial ALS and 5% of sporadic ALS cases may have *C9ORF72* mutation.^9^ Co-morbid FTD is more common in ALS patients with the *C9ORF72* genotype, and these patients may have faster disease progression and more pronounced cognitive and behavioral disorders.^9,10^

Since its discovery, there has been an intense research effort to investigate the clinical phenotypes associated with *C9ORF72* repeat expansion. More specifically, neuroimaging methods have been employed to investigate neuroanatomical features of FTD and/or ALS patients with *C9ORF72* mutation. Brain imaging may provide clinical markers for both the diagnosis and/or the follow-up of these patients, and may also shed light on the pathophysiological mechanisms of neurodegeneration associated with *C9ORF72* repeat expansion. In the current paper, we aimed to review the literature on neuroimaging studies of FTD and/or ALS patients with *C9ORF72* mutation.

## METHODS

We conducted a systematic review of the literature according to a predetermined protocol as described elsewhere.^11^ The search aimed to identify original papers reporting neuroimaging data in FTD and/or ALS patients with *C9ORF72* repeat expansion.

The search was performed in July 26^th^ 2015 on two electronic databases: PubMed and LILACS. The following terms (alone and in combination) were employed for the search on PubMed: *C9ORF72*, MRI, SPECT, PET, ALS, FTD. The same keywords were entered for the search on the LILACS database. We did not employ language or chronological filters in the search.

Titles and abstracts of the papers retrieved in the initial search were screened according to the following eligibility criteria:

[1] original research
[2] case series, cohort or cross-sectional design, and
[3] imaging methods (MRI, PET and/or SPECT). Abstracts with insufficient information, individual case reports and review articles were not included in the final selection. Disagreements on eligibility were resolved through discussion among the authors.

## RESULTS

Table 1 presents findings reported in the selected studies, including the number of patients, neuroimaging technique, and main results.

**Table 1:** Synthesis of articles included in the present review.

	Authors	Journal	Year	Population	Methods	Results
Frontotemporal dementia due to *C9ORF72* mutations	Sha et al.	Neurology	2012	• A group of patients with C9ORF72 expansion (15 bvFTD, 11 FTD-ALS and 5 ALS) was compared with 48 sporadic non-carrier patients (48 bvFTD, 19 FTD-ALS and 6 ALS)	• MRI • Analysis: VBM	The *C9ORF72* bvFTD patients showed more parietal, bilateral thalamic atrophy, compared to sporadic bvFTD patients. FTD-ALS C9ORF72 patients had more dorsal fron­tal and bilateral posterior cortical atrophy and less damage to the temporal pole than FTD-ALS sporadic patients.
Distinct clinical and pathological characteristics of frontotemporal dementia associated with *C9ORF72* mutations	Snowden et al.	Brain	2012	• 398 patients in total (221 bvFTD, 66 PNFA, 53 SD/ FTD, 68 mixed syndromes) • 32 C9ORF72- positive (19 FTD, 9 FTD/MND, 1 SD/ FTD, 3 PNFA) • Neuroimaging available for 46 C9ORF72-negative and 32 C9ORF72- positive	• MRI • CT • SPECT • Analysis: visual assessment	In the *C9ORF72*- positive group, most of the patients showed atrophy and/or hypoperfusion in frontotem­poral region. There was variability of involvement of frontal and temporal lobes, and left-right sided asym­metries.
Cognitive and clinical characteristics of patients with amyotrophic lateral sclerosis carrying a *C9ORF72* repeat expansion: a population- based cohort study	Byrne et al.	Lancet Neurology	2012	• 191 ALS cases (39 C9ORF72- positive) • Neuroimaging available for 10 patients with C9ORF72- positive and 30 patients with C9ORF72- negative	• MRI • Analysis: VBM	Significant grey-matter atrophy was found in the cohort with *C9ORF72* mutation in the right inferior frontal gyrus, right superior frontal gyrus, left anterior cingulated gyrus and the right precentral gyrus.
Clinical and pathological features of familial frontotemporal dementia caused by *C9ORF72* mutation on cromosome 9P	Hsiung et al.	Brain	2012	• 30 affected members from 16 families with the C9ORF72 mutation: -- bvFTD (n = 15) -- PNFA (n = 5) -- ALS (n = 9) -- PNFA–ALS (n = 1) • Seven subjects had final clinical diagnoses of both FTD and ALS • Neuroimaging available for 21 subjects	• CT • MRI • FDG-PET • SPECT • Analysis: visual assessment	Variable patterns were found on MRI and CT, such as focal atrophy, diffuse atrophy and normal imag­ing. PET and SPECT identified frontal abnormalities in several subjects with non-focal structural imaging. Left–right asymmetry was reported in only one case of PNFA.
Frontotemporal dementia with the *C9ORF72 * hexanucleotide repeat expansion: clinical, neuroanatomical and neuropathological features	Mahoney et al.	Brain	2012	• 273 subjects in total -- 122 bvFTD, 11 FTD-MND, 53 SD, 57 PNFA, 18 corticobasal syndrome, 11 PSPS, one Paget’s disease) • 19 C9ORF72- positive: -- 13 bvFTD, 4 FTD-MND, 2 PNFA • Neuroimaging available for 11 C9ORF72- positive	• MRI • Analysis: VBM, DTI	The mean of the brain volume was lower in the *C9ORF72 * carriers, with decreased grey matter in pre­frontal cortex and cerebellar vermis. The DTI showed increased radial diffusivity and decreased fractional anisotropy bilaterally in anterior thalamic radiations, uncinate fasciculus, anterior cingulum and ante­rior corpus callosum, right posterior corpus callosum, posterior inferior longitudinal fasciculus and superior longitudinal fasciculus.
Characterization of frontotemporal dementia and/ or amyotrophic lateral sclerosis associated with the GGGGCC repeat expansion in *C9ORF72 *	Boeve et al.	Brain	2012	• 210 bvFTD, 51 FTD/ALS, 195 ALS: -- 43 C9ORF72-positive (19 bvFTD, 11 FTD/ALS, 13 ALS) • Neuroimaging available for: MRI: 18 patients (14 bvFTD, three FTD/ALS, one ALS) • SPECT: four patients • FDG-PET: five patients	• MRI • SPECT • FDG-PET • Analysis: STAND-Maps and VBM	Neuroimaging showed bilateral frontal abnormalities most consistently, with more variable degrees of pa­rietal with or without temporal changes; no case had strikingly focal or asymmetrical findings.
Neuroimaging signatures of frontotemporal dementia genetics: *C9ORF72*, TAU, progranulin and sporadics	Whitwell et al.	Brain	2012	• 76 FTD; imaging available for: -- 19 bvFTD C9ORF72- positive -- 20 sporadic bvFTD (10 ALS)	• MRI • Analysis: VBM	The *C9ORF72* group showed symmetrical atrophy involving dorsolateral, medial and orbitofrontal lobes, and even more loss in anterior temporal lobes, pari­etal lobes, occipital lobes and cerebellum
Frontal asymmetry in behavioral variant frontotemporal dementia: clinicoimaging and pathogenetic correlates	Whitwell et al.	Neurobiology of Aging	2013	• 97 bvFTD • 11 C9ORF72- positive	• MRI • Analysis: VBM	Almost all of the *C9ORF72*- positive patients had symmetrical frontal atrophy predominantly in the temporofrontoparietal lobes.
Longitudinal neuroimaging and neuropsychological profiles of frontotemporal dementia with *C9ORF72* expansions	Mahoney et al.	Alzheimer's Research and Therapy	2012	• 20 C9ORF72- positive • Neuroimaging available for 6 C9ORF72- positive	• MRI • Analysis: volumetric measures of cortical and subcortical regions; rates of hemispheric and whole brain atrophy were calculated	Carriers exhibited a higher rate of ventricular enlargement, significant atrophy in thalamus and cerebellum and symmetrical atrophy between the cerebral hemispheres.
Cognitive decline and reduced survival in C9ORF72 expansion frontotemporal degeneration and amyotrophic lateral sclerosis	Irwin et al.	Neurology Neurosurgery Psychiatry Journal	2013	• 64 C9ORF72-positive (31 ALS, 33 FTLD) • 79 C9ORF72-negative (36 ALS, 43 FTLD) • Neuroimaging available for 41 FTLD patients (14 C9ORF72- positive and 27 C9ORF72- negative)	• MRI • Analysis: VBM	*C9ORF72* positive group had greater atrophy in the right fronto-insular, thalamus, cerebellum and bilateral parietal regions compared to *C9ORF72* negative group.
Multiparametric MRI study of als stratified for the *C9ORF72 * genotype	Bede et al.	Neurology	2013	• 39 ALS subjects: -- 9 C9ORF72-positive (6 with evidence of FTD) -- 30 C9ORF72- negative (3 with evidence of FTD) • 44 healthy controls • Neuroimaging available for all subjects	• MRI • Analysis: VMB, DTI (TBSS)	Cortical and subcortical involvement was identified in *C9ORF72* carriers, affecting fusiform, thalamic, su­pramarginal, and orbitofrontal regions. White matter abnormalities in the *sporadic * group were restricted to corticospinal and cerebellar pathways. The body of the corpus callosum and superior motor tracts were affected in both groups.
Basal ganglia involvement in amyotrophic lateral sclerosis	Bede et al.	Neurology	2013	• 39 ALS -- 9 C9ORF72- positive -- 30 C9ORF72- negative • 44 healthy controls • Neuroimaging available for all subjects	• MRI • Analysis: VBM, measures of cortical thickness, DTI (TBSS)	Compared with controls, *C9ORF72*-negative subjects had significant volume reductions in the left caudate nucleus, left hippocampus, and right accumbens nucleus. In the same comparison of groups, vertex-wise shape analyses revealed changes affecting the superior and inferior aspects of the bilateral thalami, the lateral and inferior portion of the left hippocampus, and the medial and superior aspect of the left caudate. Basal ganglia pathology was more extensive in ALS carriers.
Frontotemporal dementia associated with the *C9ORF72 * mutation: a unique clinical profile	Devenney et al.	JAMA Neurology	2014	• 114 subjects (84 FTD, 23 FTD/ALS, 7 corticobasal syndrome) • Neuroimaging • MRI: -- 10 C9ORF72-positive cases with bvFTD -- 19 C9ORF72-negative cases with bvFTD -- 35 healthy controls • FDG-PET: -- 6 C9ORF72- positive cases with normal findings on MRI	• MRI • FDG-PET • Analysis: MRI visual rating scale	A comparison of the *C9ORF72* carriers and noncar­riers confirmed no significant difference in the pre­cuneus region, but significant differences were found in the orbitofrontal cortex, anterior temporal lobe, in­sula, and anterior cingulate, with noncarriers showing greater atrophy across these regions. For 3 out 6 pa­tients, the FDG-PET showed hypometabolism in the frontal and/or temporal regions, but FDG-PET showed atypical findings for the other 3 cases.
Profiles of white matter tract pathology in frontemporal dementia	Mahoney et al.	Human Brain Mapping	2014	• 27 bvFTD (4 C9ORF72- positive) • 25 AD • 20 healthy controls • Neuroimaging available for all subjects	• MRI • Analysis: VBM, DTI (TBSS)	Widespread white matter tract pathology was identified in the bvFTD group compared with both the healthy control group and the AD group. The *C9ORF72*-positive group showed increased axial diffusivity in corpus callosum and cingulum bundle compared with controls.
Altered network connectivity in frontotemporal dementia with *C9ORF72* hexanucleotide repeat expansion	Lee et al.	Brain	2014	• 14 bvFTD C9ORF72- positive (5 MND) • 14 sporadic bvFTD (5 MND) • 14 healthy controls	• MRI • Analysis: VBM	bvFTD *C9ORF72*-positive patients showed atrophy in bilateral anterior cingulate, dorsolateral prefrontal, orbitofrontal and parietal cortices, precuneus, striatum, and bilateral thalamus. Comparing bvFTD groups, *C9ORF72*-positive patients showed greater atrophy in bilateral thalami, post central gyrus, precuneus, and parietal cortex, whereas *C9ORF72*- negative cases showed greater atrophy in bilateral anterior cingulated cortex, superior frontal gyrus, anterior insula and left striatum.
Presymptomatic cognitive and neuroanatomical changes in genetic frontotemporal dementia in the genetic frontotemporal dementia initiative (GENFI) study: a cross-sectional analysis	Rohrer et al.	Lancet Neurology	2015	• 220 subjects -- 118 C9ORF72- positive (40 symptomatic and 78 asymptomatic) -- 102 C9ORF72- negative • Neuroimaging available for 202 subjects (93 C9ORF72- negative and 109 C9ORF72- positive)	• MRI • Analysis: Volumetric analysis with cortical parcellation	The *C9ORF72*-positive group showed greater atrophy in subcortical areas including the thalamus, the insula, and posterior cortical areas. Also, differences in the frontal lobe, all subcortical volume, and whole-brain volume were noted between carriers and non-carriers at 5 years before expected onset.
Brain atrophy over time in genetic and sporadic frontotemporal dementia: a study of 198 serial magnetic resonance images	Whitwell et al.	European Journal of Neurology	2015	• 58 subjects -- 11 C9ORF72-positive -- 15 sporadic FTD • Neuroimaging available for all subjects	• MRI • Analysis: rates of regional, lobar and global atrophy were calculated	Progressive brain atrophy was observed in all groups, but greatest rates of atrophy were found in the frontal and temporal lobes. Sporadic FTD showed greater rates of atrophy in the anterior cingulate than the *C9ORF72*-positive group.
Value of 18fluorodeoxyglucose-positron-emission tomography in amyotrophic lateral sclerosis: a prospective study.	Van Laere et al.	JAMA Neurology	2014	• 70 ALS patients -- 11 C9ORF72-positive -- 59 C9ORF72-negative • Healthy controls • Neuroimaging available for all subjects	• FDG-PET • MRI • Analysis: VBM	PET showed perirolandic and variable prefrontal hypometabolism in most patients. *C9ORF72*- positive ALS patients had discrete hypometabolism in the thalamus and posterior cingulate compared with *C9ORF72*-negative individuals. Extensive hypometabolism in the prefrontal or anterior temporal areas was present in a few patients and associated with significantly shorter survival.
The metabolic signature of *C9ORF72*-related ALS: FDG PET comparison with nonmutated patients	Cistaro et al	European Journal of Nuclear Medicine and Molecular Imaging.	2014	• 15 ALS C9ORF72-positive • 12 ALS-FTD C9ORF72- negative • 30 ALS C9ORF72- negative • Neuroimaging available for all subjects	• PET-FDG • Analysis: VBM	The ALS *C9ORF72*-positive cases compared with the patients without mutations of ALS-related genes showed significant hypometabolism in the anterior and posterior cingulate cortex, insula, caudate and thalamus, the left frontal and superior temporal cortex. The ALS *C9ORF72*-positive patients showed hypometabolism in the left temporal cortex compared with the ALS-FTD patients.
The phenotype of the *C9ORF72* expansion Carriers according to revised criteria for bvFTD	Solje et al	PLoS ONE	2015	• 32 bvFTD • 4 bvFTD-ALS • All cases were carriers • Neuroimaging available for all subjects: • MRI: 17, CT: 7, both MRI and CT: 12, SPECT or PET: 17	• MRI and • CT • PET and • SPECT Analysis: visual assessment	Diffuse cortical and central atrophy without frontal or temporal predominance was detected in eight cases. Two cases had normal brain MRI imaging. PET/SPECT was normal in 17.6% of patients with bvFTD.

AD: Alzheimer's disease; ALS: amyotrophic lateral sclerosis; bvFTD: behavioral variant frontotemporal dementia; CT: computed tomography; DTI: diffusion tensor imaging; FTD: frontotemporal dementia; FTLD: frontotemporal lobar degeneration; FTLD-TDP: frontotemporal lobar degeneration with TDP-43 pathology; MND: motor neuron disease; MRI: magnetic resonance imaging; PET: positron emission tomography; PNFA: progressive non-fluent aphasia; PSPS: progressive supranuclear palsy syndrome; SD/FTD: semantic dementia with frontal features; SPECT: single photon emission computed tomography; -Maps (STructural Abnormality due to NeuroDegeneration-Maps); TBSS: tract-based spatial statistics; TBM: tensor based morphometry; VBM: voxel based morphometry

**Figure 1:**
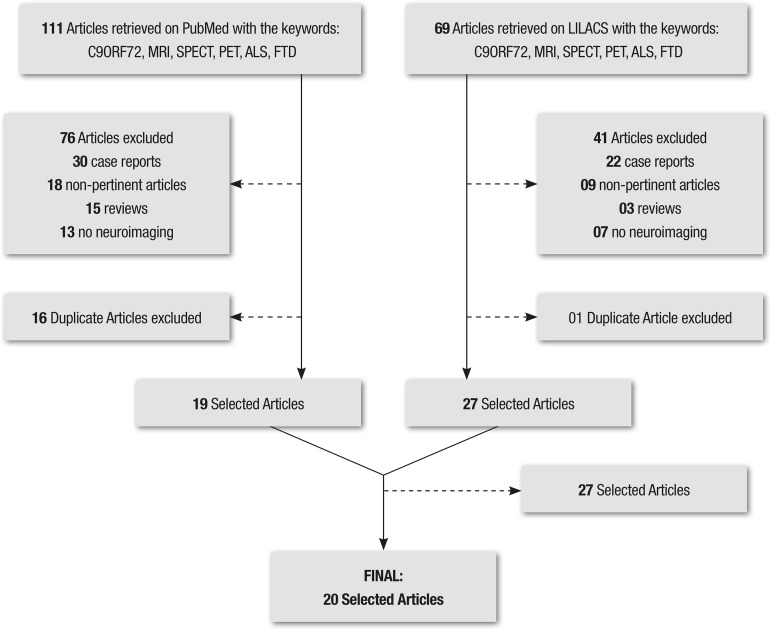
Flowchart depicting selection of items for systematic review on PubMed and Lilacs databases using the terms C9ORF72, ALS, FTD, MRI, SPECT and PET.

The initial search resulted in 110 and 69 papers retrieved on PubMed and LILACS, respectively. After this initial screening, papers were selected according to the aforementioned inclusion criteria and duplicate articles removed. The final selection comprised twenty articles (Figure 1).

Selected publications are presented below in three parts: Part I, comprising studies which included FTD patients only; Part II, which describes studies limited to ALS patients; and Part III, which presents studies that included ALS, FTD and FTD-ALS patients.

**Part I: FTD Patients.** A series of studies assessed the pattern of brain atrophy in FTD patients with *C9ORF72*^10,12-21^ using mainly MRI volumetric analysis.

A widespread, symmetrical pattern of brain atrophy was reported in FTD-*C9ORF72* patients compared with healthy controls.^12,13^ The more atrophic compromised areas were the anterior brain regions, including temporal lobes and all the main subregions of the prefrontal cortex (dorsolateral, orbitofrontal and medial regions). Atrophy in posterior regions (parietal and occipital regions) was also observed in *C9ORF72* carriers.^13,14^ However, these findings were not replicated in a series of *C9ORF72* FTD patients in which brain atrophy was assessed using a visual rating scale, and which failed to find significant differences in atrophy patterns between carriers and healthy controls in prefrontal regions (orbitofrontal cortex, anterior cingulate) or temporal regions.^16^ A recent study reported that carriers of *C9ORF72* repeat expansion exhibited significant atrophy in specific brain regions in the pre-symptomatic phase of FTD (before the onset of clinical symptoms).^19^ Compared to healthy controls, *C9ORF72* carriers had marked atrophy in subcortical (thalamus, e.g.) and cortical regions (including frontal, temporal and parietal regions) 20-25 years prior to expected disease onset.^19^

Investigating white matter tract changes in different genetic groups of bvFTD^21^ compared with healthy controls, *C9ORF72* carriers had altered diffusivity in the corpus callosum and cingulum bundle. However, these data are limited by the small size of the sample (only four bvFTD carriers).

Some studies compared neuroimaging features of *C9ORF72-*bvFTD with sporadic bvFTD and other mutations. *C9ORF72-*bvFTD patients had less gray matter loss than sporadic bvFTD in the anterior cingulate, orbitofrontal cortex, anterior temporal lobe and insula.^16^ Another study reported that the majority of subjects with mutation in the microtubule associated protein tau gene (*MAPT*) and *C9ORF72* subjects had symmetric frontal atrophy, while most subjects with mutation in the progranulin gene (*GRN*) had asymmetric atrophy.^13^ *C9ORF72* carriers had greater atrophy in posterior (parietal and occipital) lobes in comparison with *MAPT* and sporadic bvFTD groups, while patients with *MAPT* mutations had greater impairment in temporal poles, compared with the *C9ORF72* group.^13^ Patients with *GRN* mutation had more loss in parietal lobes than *C9ORF72* carriers.^13^ In the same study, by applying a multinomial logistic regression model based on atrophic patterns, it was possible to classify FTD patients with different genotypes with 93% accuracy, suggesting that neuroimaging may be useful to distinguish *C9ORF72*-FTD patients from patients with other mutations at a single-subject level.^13^

Only one study investigated white matter patterns across bvFTD patients grouped according to genetic status.^21^ There were no differences between *C9ORF72* and sporadic bvFTD cases, but *MAPT* patients had abnormal fractional anisotropy in the anterior region of the left temporal lobe, compared with the *C9ORF72* group.

The integrity of the intrinsic connectivity network in bvFTD was explored in a group of 14 bvFTD *C9ORF72* carriers and 14 bvFTD non-carriers.^12^ These groups were compared against healthy controls. Patients with *C9ORF72* did not exhibit differences in the default mode network compared to controls. Conversely, bvFTD non-carriers exhibited a different pattern, presenting both impaired (in striatum and thalamus) and enhanced (in precuneus and posterior cingulate) connectivity compared with controls.^12^ In the same study, it was reported that *C9ORF72* carriers had impaired sensorimotor connectivity in striatum and thalami, compared with bvFTD non-carriers. There was no difference in the salience network connectivity between carriers and non-carriers.^12^

The progression of brain atrophy in bvFTD patients with different genetic status was assessed in a longitudinal study.^18^ *GRN* patients had greater rates of atrophy than sporadic, *MAPT* and *C9ORF72* groups. Sporadic bvFTD patients had greater rates of gray matter loss in anterior cingulate than *C9ORF72* carriers, while the latter had greater rates of atrophy in cerebellum and occipital lobes, compared with *MAPT* carriers.^18^ Another study found that *C9ORF72-*bvFTD patients had increased rates of brain atrophy and ventricular expansion compared with healthy controls.^14^

In summary, widespread brain atrophy was reported in FTD *C9ORF72* patients, mostly in anterior brain regions, but also with possible damage in posterior cortical areas. Brain atrophy may be identified before disease onset. However, the absence of significant changes in FTD *C9ORF72* carriers has also been reported.

**Part II: ALS Patients.** Five articles investigated neuroimaging features of ALS patients with *C9ORF72* expansion. Four of these studies employed the MRI technique^9,22-24^ while the remainder used FDG-PET.^25^

ALS patients with *C9ORF72* mutation had greater atrophy in prefrontal regions, including frontal gyri and the anterior cingulate, compared to those with sporadic ALS.^9,22^ The right precentral gyrus was also affected in one study.^9^ Mild hypometabolism in the thalamus and posterior cingulate was found on PET-FDG in ALS carriers compared with non-carriers.^25^ Compared with ALS non-carriers, *C9ORF72* carriers had more cortical and subcortical involvement, affecting both cortical (fusiform, supramarginal, and orbitofrontal cortex and Broca's area) and subcortical regions (thalamus).^23^ Interestingly, in the same study, white matter abnormalities in ALS non-carriers were relatively limited to corticospinal and cerebellar pathways, while carriers had more widespread involvement. These data suggested that non-motor changes (e.g. cognitive impairment) in ALS could be largely driven by *C9ORF72* repeat expansion.^23^ Basal ganglia involvement was also more extensive in ALS patients with *C9ORF72* mutation than in non-carriers.^24^

In short, ALS *C9ORF72* carriers had greater atrophy, with predominance in prefrontal regions, compared to sporadic ALS patients. Mild hypometabolism in the thalamus and posterior cingulate, more widespread abnormalities of white matter, and greater basal ganglia involvement has also been demonstrated in ALS carriers compared with non-carriers.

**Part III: FTD, ALS and FTD-ALS patients.** The imaging patterns of patients with ALS, FTD or FTD-ALS according to their genetic status were compared in a series of studies.^5,10,20,22,26,27^ Most of the papers employed structural brain MRI.

In a group of eighteen patients with *C9ORF72* repeat expansion (fourteen bvFTD, three with FTD/ALS and one with ALS), gray matter loss was found in cortical areas including frontotemporal regions,^26^ in a similar pattern to that reported by others.^10,20,22^ Most studies reported symmetrical patterns of brain atrophy, except for patients presenting with predominant language deficit. Some patients may have parietal cortical atrophy and thalamic involvement.^10,26^ These studies are limited by the absence of direct comparisons between bvFTD and FTD-ALS.

A group of patients with C9ORF72 expansion (15 bvFTD, 11 FTD-ALS and 5 ALS) was compared against 48 sporadic non-carrier patients (48 bvFTD, 19 FTD-ALS and 6 ALS).^27^ The authors found that bvFTD-C9 patients had more parietal and bilateral thalamic atrophy and less medial frontal atrophy compared to sporadic bvFTD patients. FTD-ALS C9ORF72 patients had more dorsal frontal and bilateral posterior cortical atrophy and less damage to the temporal pole than sporadic FTD-ALS patients.^27^

Conversely, some studies reported that *C9ORF72* carriers may not have brain atrophy.^22,26^ These findings were expanded by a recent study, which demonstrated that almost 18% of bvFTD cases with *C9ORF72* mutation had no abnormalities on PET/SPECT.^5^

In a study that investigated the metabolic patterns of *C9ORF72* carriers on PET-FDG, ALS carriers of *C9ORF72* had more pronounced hypometabolism in cortical (cingulate cortex, and frontotemporal regions) and subcortical structures (caudate and thalami) compared with sporadic ALS patients.^28^ In the same study, ALS patients with *C9ORF72* expansion had impaired metabolism in the left temporal cortex, compared with the ALS-FTD group.^28^ Accordingly, ALS *C9ORF72* patients may have a more severe clinical picture and more widespread central nervous system involvement than sporadic ALS patients, regardless of the association with bvFTD.

Taken together, *C9ORF72* carriers had symmetrical gray matter loss in cortical regions, except for patients with predominant language deficit, who demonstrated asymmetrical cortical involvement. ALS *C9ORF72* patients had more widespread central nervous system involvement than sporadic ALS and/or FTD groups. Some studies have reported an absence of abnormalities on structural and functional neuroimaging.

## DISCUSSION

For many years, neuroimaging was of limited applicability in the everyday evaluation of neurodegenerative disorders. For instance, the exclusion of focal lesions or hydrocephalus as causes of cognitive deficits was the main utility of imaging exploration in patients suffering from cognitive disorders. This picture has changed, with modern imaging techniques which provide useful and specific markers for the diagnosis and the follow-up of neurodegenerative diseases, such as ALS and FTD.^29^ In this paper we systematically reviewed neuroimaging data in FTD and/or ALS patients with *C9ORF72* repeat expansion.

Most studies that investigated the neuroimaging features of *C9ORF72* carriers found consistent involvement of frontotemporal regions, including prefrontal cortex, (dorsolateral, orbitofrontal and medial regions), and also cingulate and posterior regions such as the parietal and occipital lobes.^10,12-15^ Subcortical regions, especially thalami, may also be affected in *C9ORF72* carriers.^10,19,22^ It is of note that some studies reported that patients with *C9ORF72* mutation may not have abnormalities on structural and functional brain imaging.^5,16,22,26^ These disparate patterns may be due to a number of different reasons. The inclusion of patients at different stages of disease and differences in neuroimaging methods across studies may account for the variability of results. One factor that may partially account for these disparate findings is that different phenotypes are associated with *C9ORF72* and heterogeneity may occur even among patients with the same clinical phenotype.^22^ Besides ALS, bvFTD and ALS/FTD, *C9ORF72* mutation has also been associated with primary progressive aphasia, Huntington's disease-like syndrome, and atypical parkinsonism syndromes, such as corticobasal degeneration and progressive supranuclear palsy.^22,30-33^ Repeated expansion in *C9ORF72* may also contribute to Alzheimer's disease.^33,34^ In summary, although FTD and/or ALS are the most common phenotypes of *C9ORF72* repeat expansion, other clinical presentations may occur, with different neuroimaging patterns. It remains unclear why some patients with the C9ORF72 expansion have minimal atrophy on neuroimaging studies. The possible pathways by which *C9ORF72* mutation participates in the pathophysiological process associated with different neurodegenerative diseases also remain elusive.

From a clinical perspective, the variability of clinical findings associated with *C9ORF72* limits the interpretation of neuroimaging features at an individual level. A single-center study reported the utility of a multinomial regression model to accurately identify *C9ORF72* patients based on patterns of brain atrophy^13^ at single-subject level. However, this strategy seems limited to research centers with advanced expertise in neuroimaging techniques. Moreover, *C9ORF72* carriers may have no structural abnormalities on brain MRI.^16,22,26^ Therefore, atrophic features in brain MRI are of limited value for identification of *C9ORF72* carriers in clinical practice.

On the other hand, neuroimaging assessment may be useful for the follow-up of patients with *C9ORF72* repeat expansion and for suggesting prognostic aspects. FTD and/or ALS patients with *C9ORF72* mutation may have faster disease progression and shorter survival than non-carriers,^10,16^ even though this is not consistent across studies.^27^ In this scenario, neuroimaging can identify markers of disease progression, such as the rate of brain atrophy and ventricular expansion.^14^ These markers could help track disease changes and guide clinical management, especially in the prospect of disease-modifying drugs that will target the pathophysiological process of neurodegenerative disorders.

New modern neuroimaging techniques may provide useful biomarkers for the diagnosis and follow-up of *C9ORF72* carriers. Disruption of functional connectivity may be seen in the absence of brain atrophy and could be regarded as an early marker of disease.^12^ Only one study to date explored functional connectivity in *C9ORF72* carriers, and found that there is a convergent, large-scale, disrupted network among different patterns of brain atrophy.^12^ The investigation of functional connectivity may enhance our understanding about the neural networks compromised by *C9ORF72* mutation, thus providing valuable information for the comprehension of the pathophysiology of the FTD-ALS spectrum.

Techniques exploring the integrity of the white matter tract may also be of clinical value in the assessment of patients with *C9ORF72* repeat expansion. Degeneration of the corticospinal tract is a hallmark of ALS, and disruption of this tract can differentiate ALS patients from bvFTD and ALS-FTD patients.^23,35^ Further studies are needed to describe putative white matter changes associated with*C9ORF72* mutation.

Besides its value for diagnostic purposes, neuroimaging is also important for the understanding of the neural basis of cognitive and behavioral disorders observed in the FTD-ALS spectrum. ALS and bvFTD patients with *C9ORF72* mutation have a greater frequency of psychiatric disorders, especially psychotic symptoms, such as delusions, paranoid ideation and hallucinations.^5,16,22,36^ Indeed, almost 40% of FTD patients with *C9ORF72* repeat expansion presented psychotic symptoms.^22^ Paranoid or irrational thinking were also frequent in the same study.^22^ The neuropsychological profile of bvFTD patients with *C9ORF72* expansion is similar to non-carrier bvFTD patients,^16^ with comparable performance in memory, language and executive skills. Deficits in executive functions are the most common observed feature in ALS-FTD patients^3^ and can also be associated with prefrontal dysfunction.

Taken together, these data emphasize the complex interaction between *C9ORF72* mutation and clinical presentations of neurodegenerative diseases, especially the FTD-ALS spectrum. The discovery of the *C9ORF72* repeat expansion has opened a window for the understanding of the continuum between FTD and ALS. The next advances in neuroimaging investigation may provide valuable markers for the diagnosis and follow-up of these patients, and may also clarify the common pathophysiological pathways between ALS and FTD, with possible clinical outcomes.
